# Happy Is the Wooing That Is Not Long Adoing

**Published:** 1854-12

**Authors:** 


Happy is the Wooing that is not Long Adoing.—An eminent writer says: "It is my firm opinion, derived from experience, that the period of courtship cannot be too short. I have reason to say, that when you have hooked your fish, the sooner you use your landing net the better."

